# Psychedelic Safety and Clinical Outcomes Across Exposure Patterns: A Structured Narrative Review

**DOI:** 10.3390/life16081338

**Published:** 2026-08-14

**Authors:** Szabolcs Kéri

**Affiliations:** 1 Sztárai Institute, Tokaj-Hegyalja University, 3950 Sárospatak, Hungary; keri.szabolcs@unithe.hu; 2 Department of Physiology, Albert Szent-Györgyi Medical School, University of Szeged, 6720 Szeged, Hungary

**Keywords:** psilocybin, MDMA, LSD, adverse events, functional unblinding, microdosing, exposure frequency, hallucinogen persisting perception disorder, 5-HT2B (serotonin 2B) receptor

## Abstract

Clinical trials and naturalistic studies describe psychedelics under markedly different exposure conditions. This narrative review searched PubMed/MEDLINE, the Cochrane Library, ClinicalTrials.gov, and regulatory sources through 15 July 2026, and examined safety and outcomes across four dimensions of exposure: dose, frequency, person, and context. In screened adults receiving one to three supervised doses, transient headache, nausea, anxiety, and cardiovascular activation are common; serious reactions are uncommon but include persistent anxiety, suicidality signals, and a recent case of hallucinogen persisting perception disorder. Evidence supports MDMA (3,4-Methylenedioxymethamphetamine) -assisted therapy for post-traumatic stress disorder and psilocybin-based interventions for depression, although functional unblinding, selected samples, inconsistent comparators, and a recent negative primary trial constrain certainty. Naturalistic studies suggest that vulnerability and context modify outcomes, but frequency is inconsistently measured and rarely linked to cumulative exposure or long-term health. Short-term microdosing trials show limited benefit, whereas long-term cardiac and psychiatric safety remains unresolved. Exposure-defined cohorts, prospective registries, echocardiographic surveillance, and blinding-aware comparative trials are priorities.

## 1. Introduction

The clinical development of psychedelics has accelerated from small experimental studies to multicenter randomized trials, phase 3 programs, and limited real-world access. Classic serotonergic psychedelics, including psilocybin, lysergic acid diethylamide (LSD), N,N-dimethyltryptamine (DMT), ayahuasca, and mescaline, share prominent agonist activity at the serotonin 2A receptor but differ substantially in pharmacokinetics, receptor profiles, and duration of action. 3,4-Methylenedioxymethamphetamine (MDMA) is an entactogen rather than a classic psychedelic and should be analyzed separately whenever its pharmacology or exposure pattern matters [1,2].

The emerging evidence is divided between two settings. Clinical trials generally administer one to three known doses to screened participants, with preparation, continuous monitoring, and post-session integration. Naturalistic studies include more heterogeneous users, compounds, doses, motives, co-exposures, and environments, but usually rely on self-selection and self-report. Consequently, the two literatures do not estimate the same causal quantity. A low rate of severe complications in a screened, supported trial cannot be assumed to apply to frequent unsupervised use; conversely, harms reported after unverified polydrug exposure cannot be attributed directly to a pharmaceutical-grade compound administered twice in a controlled setting [3,4].

Regulatory developments make this distinction clinically important. Australia has permitted authorized psychiatrists to prescribe MDMA for post-traumatic stress disorder (PTSD) and psilocybin for treatment-resistant depression since July 2023, subject to ethics and regulatory controls. These products remain unapproved therapeutic goods rather than medicines that have undergone the ordinary marketing authorization assessment [5,6]. Two phase 3 trials of MDMA-assisted therapy reported substantial improvements in PTSD, whereas a large phase 2b psilocybin trial for treatment-resistant depression recently failed to meet its primary endpoint [7,8,9]. These developments reinforce the need to separate promising findings from established evidence.

This review integrates supervised clinical trials, naturalistic studies, population analyses, and mechanistic evidence using an exposure-based framework. The central questions are: (1) which adverse events are reliably observed under supervised administration; (2) how strong and durable are the clinical outcome signals; (3) how do functional unblinding and study design alter interpretation; and (4) what is known about repeated use, including microdosing? The purpose is not to classify psychedelics as intrinsically safe or unsafe, but to identify the exposure conditions under which particular claims are supported.

## 2. Methods

### 2.1. Review Design and Reporting Approach

A structured narrative review design was chosen because the review question spans heterogeneous interventions, study designs, outcomes, and exposure settings that are not amenable to a single pooled estimate. The review follows the principles of the SANRA instrument for narrative reviews: explicit justification of the review, a reproducible search strategy, critical appraisal, and claim-to-source matching [10].

### 2.2. Information Sources and Search Strategy

PubMed/MEDLINE, the Cochrane Library, and ClinicalTrials.gov were searched for English-language human studies published from 1 January 2000 through 15 July 2026. Earlier literature was considered for historically important safety series. Searches were supplemented by backward and forward citation chaining, targeted searches of major journals, and official regulatory sources from the US Food and Drug Administration (FDA) and the Australian Therapeutic Goods Administration (TGA). Company announcements were included only when they contained consequential late-phase results not yet available in peer-reviewed form, and they are explicitly labeled as provisional.

The core search combined compound terms (psilocybin OR psilocin OR LSD OR “lysergic acid diethylamide” OR DMT OR dimethyltryptamine OR ayahuasca OR mescaline OR MDMA OR midomafetamine) with outcome and design terms (“adverse event*” OR safety OR suicid* OR psychosis OR mania OR “hallucinogen persisting perception disorder” OR HPPD OR cardiovascular OR efficacy OR depression OR PTSD OR anxiety OR “substance use disorder” OR follow-up OR durability OR unblind* OR expectancy OR frequency OR repeated OR microdos* OR “5-HT2B” OR valv*). A supplementary frequency search combined the compound terms with (regular OR frequent OR monthly OR repeated OR cumulative OR lifetime OR long-term OR maintenance OR microdosing) and with mental-health, cardiovascular, neurocognitive, and functional outcomes.

### 2.3. Eligibility and Evidence Selection

**Figure 1:**
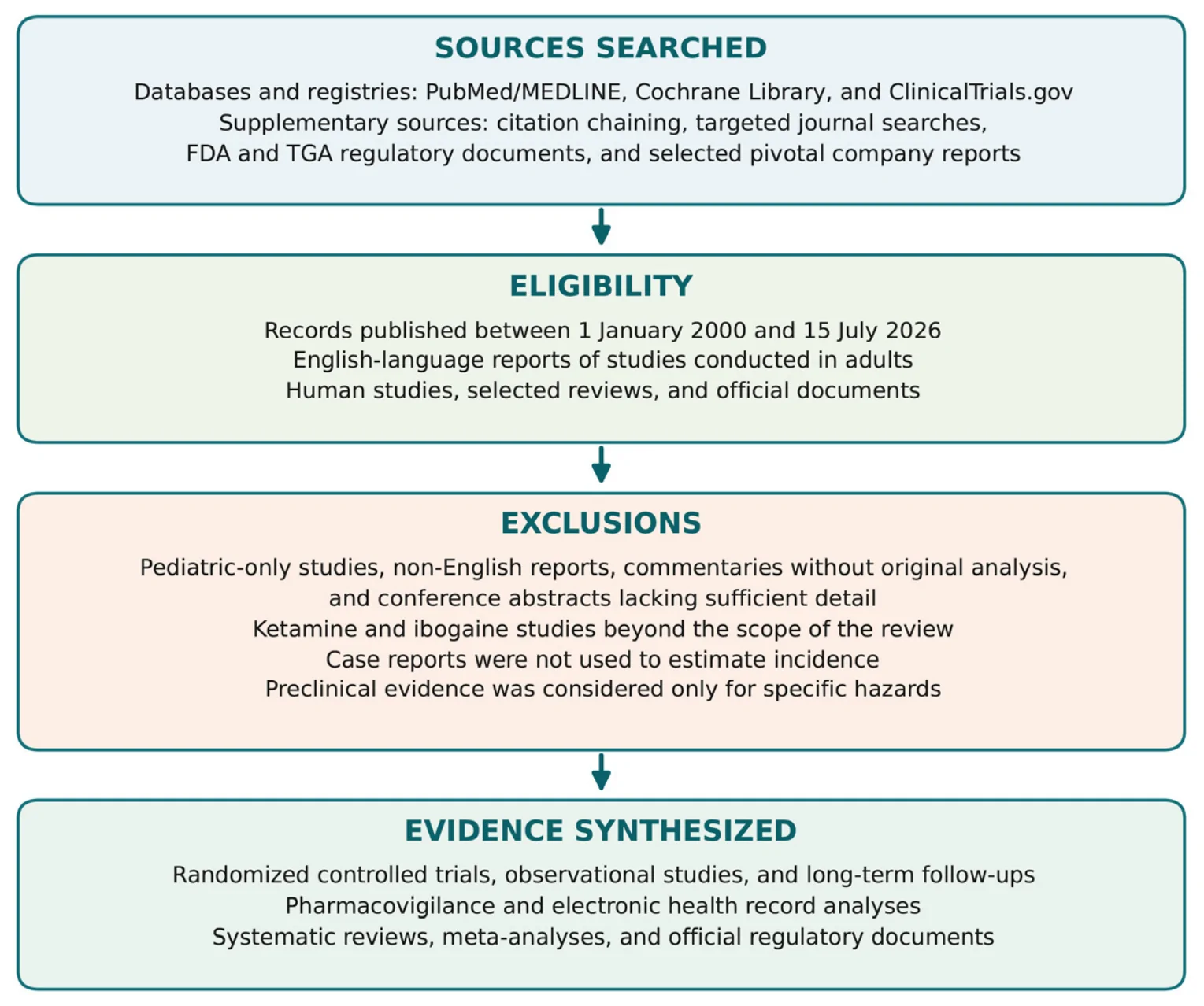
Illustration of study selection and eligibility.

4823 records were identified: 4702 through the database and registry searches and 121 through citation chaining, targeted searches, and regulatory or sponsor sources. After removal of duplicates, 3820 records were screened. 241 reports were assessed in full text. 84 reports were included in the narrative review (Figure 1).

### 2.4. Quality Appraisal and Synthesis

Evidence was appraised by design. Randomized trials were assessed using the RoB 2 domains, with particular attention to allocation, missing outcome data, outcome selection, and compromised blinding. Non-randomized intervention and exposure studies were evaluated using ROBINS-I principles, especially confounding, selection, exposure misclassification, and outcome ascertainment. Systematic reviews were judged against AMSTAR 2 domains, including protocol specification, search completeness, duplicate processes, risk-of-bias assessment, and interpretation [11,12,13]. No composite quality score was calculated. Instead, conclusions were qualitatively calibrated as more or less credible based on risk of bias, directness, consistency, precision, adverse-event ascertainment, and whether the reported comparison addressed the clinical question.

The review was conducted by a single author. Study selection, extraction, and appraisal were independently duplicated by an expert who did not participate in the review.

## 3. Compound-Specific Exposure

Compound identity is necessary but not sufficient for interpreting risk or benefit. Each exposure should also be indexed by four interacting dimensions: dose, frequency, person, and context. The framework is not intended as a validated prediction rule. Its value is conceptual: it prevents evidence generated in one exposure pattern from being transferred uncritically to another.

**Figure 2:**
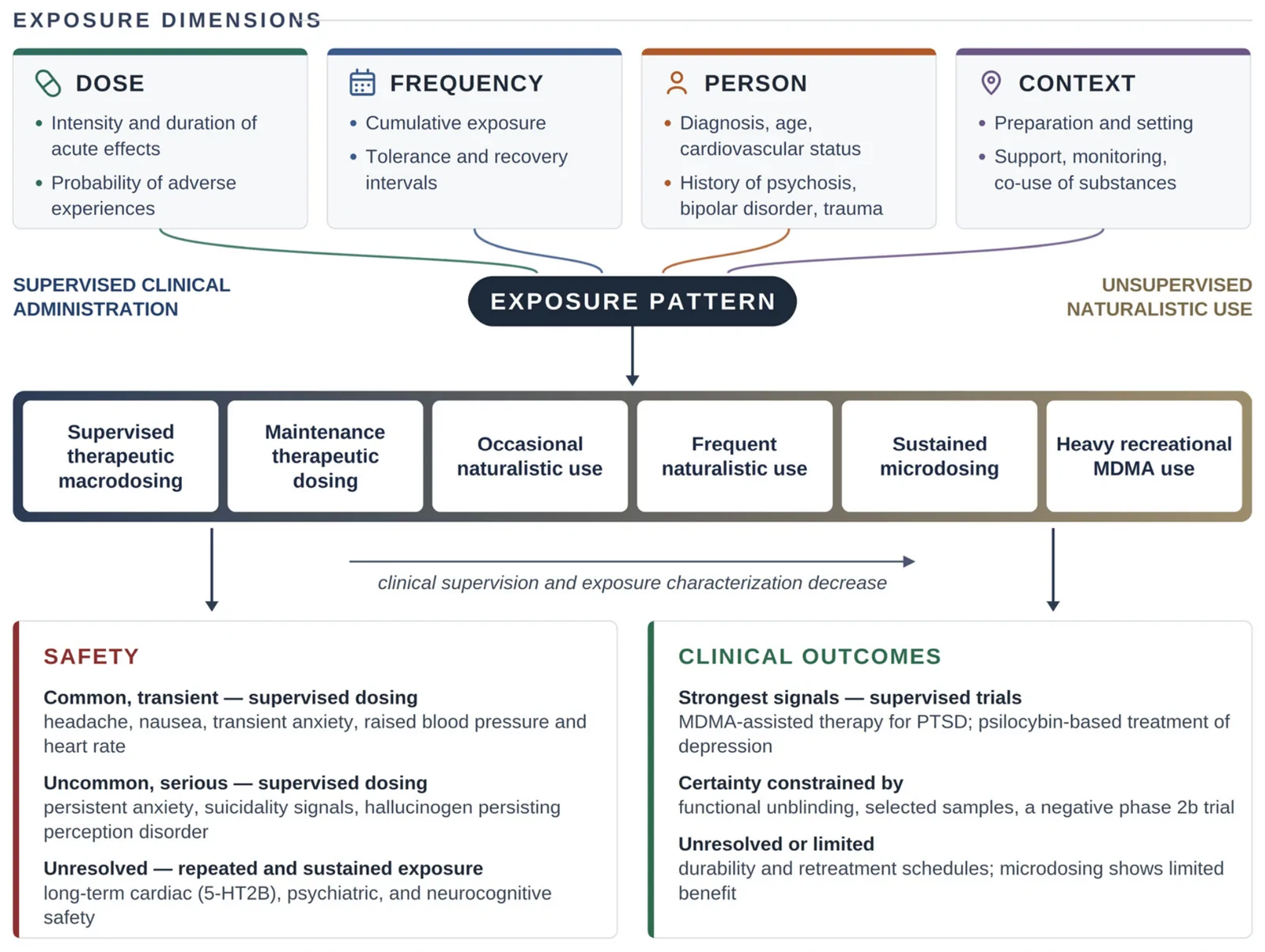
The four-dimensional exposure framework and its relationship with safety and clinical outcomes. Dose, frequency, person, and context jointly define the exposure pattern under which classic psychedelics and MDMA are used. Principal exposure patterns (Table 1) are arranged schematically along a continuum from supervised clinical administration to naturalistic use. The exposure pattern, rather than compound identity alone, determines which safety findings and which clinical outcomes are supported by the available evidence. Abbreviations: 5-HT2B, serotonin 2B receptor; MDMA, 3,4-methylenedioxymethamphetamine; PTSD, post-traumatic stress disorder.

Dose determines the intensity and duration of acute effects, physiological activation, and the probability of an adverse experience. Frequency determines cumulative exposure, opportunities for recovery, tolerance, and the relevance of chronic receptor stimulation. Person includes diagnosis, age, cardiovascular status, personal or family history of psychosis or bipolar disorder, concurrent medication, trauma history, and prior drug experience. Context includes preparation, physical setting, social support, therapeutic alliance, monitor competence, co-use of other substances, and follow-up. Therapeutic alliance and characteristics of the acute experience correlate with clinical outcome, although these associations are partly confounded and should not be interpreted as proof of mediation [14,15]. The principal exposure patterns and their associated uncertainties are summarized in Table 1 and Figure 2.

The modern therapeutic literature is characterized by a limited number of known doses administered to screened participants in supportive settings. Naturalistic use covers a much larger and less measurable space. Microdosing is not an evidence-free practice, but it remains poorly characterized as a sustained exposure because most trials last only weeks. The key gap is therefore not simply long-term psychedelic safety; it is long-term safety and effectiveness under clearly defined repeated-exposure patterns.

## 4. Safety Under Supervised Administration

### 4.1. Acute Adverse Events and Physiological Activation

In contemporary trials, the most consistent acute adverse events with classic psychedelics are headache, nausea, transient anxiety or fear, dizziness, fatigue, and increases in blood pressure and heart rate. MDMA is additionally associated with jaw clenching, muscle tightness, reduced appetite, sweating, chills, and sleep disturbance. Meta-analyses generally characterize these events as common, predominantly mild to moderate, and time-limited, while emphasizing substantial heterogeneity in definitions and ascertainment [16,17,18,19].

Incidence figures must be read in light of how the events were elicited. Spontaneous reporting yields lower rates than structured checklists, and some studies record expected subjective or autonomic drug effects as adverse events while others do not. Expectedness does not make an event clinically irrelevant: a foreseeable increase in blood pressure may still matter in an older participant or a person with vascular disease. Severity, duration, intervention, and functional consequence are therefore more informative than a binary event count. Reviews of psilocybin trials have repeatedly identified inconsistent adverse-event definitions, incomplete reporting windows, and high risk of bias related to blinding [20,21].

Clinical intervention during dosing is uncommon but documented. Benzodiazepines have occasionally been used for persistent anxiety, antihypertensives for marked blood-pressure elevation, and routine symptomatic medication for nausea or headache. In an older cohort of long-term AIDS survivors, moderate or severe hypertension occurred more often than in younger samples, illustrating that physiological risk is modified by age and baseline health [22]. Two participants in a psilocybin study conducted during concomitant selective serotonin reuptake inhibitor treatment required clonidine for severe blood-pressure elevation [23]. In a small post-treatment Lyme disease pilot, hypertension and headache were frequent but resolved without serious sequelae [24].

### 4.2. Serious Adverse Events, Suicidality, and Psychiatric Deterioration

Serious adverse events are uncommon in supervised trials, but the precision of incidence estimates remains limited. Systematic reviews include only a few thousand exposed participants, use different causality rules, and often combine healthy volunteers with patients who have substantial baseline psychiatric risk [16,17]. The best-supported conclusion is therefore that contemporary screened trials have not revealed a frequent, severe toxicity syndrome. This is not the same as showing that serious harm is impossible or that its incidence is known.

Suicidality requires event-level interpretation. In the two pivotal MDMA-assisted therapy trials, suicidality was monitored in populations with severe PTSD; some serious suicidal events occurred in comparator conditions, and group-level PTSD improvement was accompanied by overall reductions in symptom burden [7,8]. Individual-participant meta-analysis in psychedelic trials found reductions in suicidality on average, yet a minority experienced acute or post-acute increases [25]. There were two omitted suicidality-related events (one suicide after placebo-like 1-mg psilocybin and one later attempt after active treatment) [26]. Similarly, symptom worsening after psilocybin occurred in a clinically meaningful minority but at rates comparable with an escitalopram comparator in the available participant-level synthesis [27]. Group-level benefit therefore does not eliminate idiosyncratic deterioration.

Several newer trials caution against an overly reassuring account. In a randomized LSD trial for major depression, three participants (two in the low-dose group and one in the high-dose group) were hospitalized for worsening depression 24 to 102 days after dosing; alternative explanations were present, and investigators judged an association with LSD unlikely. A participant from the low-dose group died by suicide seven months after withdrawing from the study; the event was not recorded as a study serious adverse event and was not attributed to LSD [28]. A 2026 randomized psilocybin study reported persistent severe anxiety requiring medical attention in two participants [29]. The EPISODE trial reported a higher frequency of dosing-day suicidal ideation in the 25-mg group and two serious adverse reactions, including one case of HPPD [9]. These events are rare, but they demonstrate that serious psychiatric adverse reactions can occur.

Persistent psychosis has not emerged in contemporary trials, but participants with personal or family histories of psychotic or bipolar disorders are usually excluded. Historical clinical series, conducted under less standardized screening and support, reported prolonged psychotic reactions after LSD or mescaline in a small minority [30,31]. The contrast is consistent with the protective value of screening and structured care; it does not establish safety in populations intentionally omitted from the evidence base.

### 4.3. Delayed Effects and Hallucinogen Persisting Perception Disorder (HPPD)

Long-term follow-up after supervised dosing is generally reassuring but methodologically limited. Twelve-month follow-up after LSD-assisted therapy and psilocybin treatment has not identified a consistent pattern of delayed toxicity, and small multi-year follow-ups describe sustained improvement without drug dependence [32,33,34,35]. These cohorts are small, often uncontrolled, vulnerable to selective retention, and not powered to detect rare outcomes. They support the absence of a common late toxicity syndrome after one or a few supervised doses, but they do not quantify very rare risks.

HPPD illustrates the importance of outcome definition. A prospective naturalistic cohort found that 32.1% of participants reported at least one visual phenomenon resembling an HPPD symptom four weeks after psychedelic use, most commonly intensified color, afterimages, or peripheral movement, but fewer than 1% found these effects distressing. Younger age, female sex, psychiatric history, and trait absorption predicted symptoms [36]. Because clinically significant HPPD requires distress or functional impairment, subclinical visual after-effects should not be equated with the disorder.

At the other end of the ascertainment spectrum, a large electronic-health-record analysis identified 25,778 people with an HPPD diagnosis and substantial psychiatric, headache, and pain comorbidity [37]. Case series also describe heterogeneous presentations, frequent polydrug exposure, overlap with anxiety and visual snow syndrome, and uncertain temporal attribution [38,39,40]. The recent treatment-related HPPD case in means that “zero cases in modern trials” is no longer accurate. Non-distressing visual after-effects are relatively common in naturalistic users, clinically impairing HPPD is uncommon, and its incidence after supervised therapeutic administration remains uncertain.

## 5. Clinical Outcomes and Durability

### 5.1. PTSD and MDMA-Assisted Therapy

The strongest late-phase therapeutic signal is for MDMA-assisted therapy in PTSD. Two phase 3 trials reported large within-group improvements and clinically important between-group advantages in PTSD severity and functional impairment after three MDMA sessions integrated into a manualized psychotherapy program [7,8]. Meta-analytic syntheses support efficacy but lower the certainty of that conclusion because of functional unblinding, the small number of studies, overlapping investigators and sites, and incomplete adverse-event reporting [19,41]. The intervention should be described as MDMA-assisted therapy rather than MDMA alone because the drug, psychotherapy, preparation, and monitoring were delivered as a package.

The US regulatory outcome tempers, but does not erase, the efficacy signal. The FDA issued a complete response letter in August 2024 requesting an additional adequate and well-controlled trial and identifying concerns regarding benefit durability, safety characterization, prior MDMA exposure, and trial conduct; the letter became public in September 2025 [42]. Regulatory non-approval is not equivalent to proof of inefficacy. It indicates that the submitted evidence did not meet the agency’s standard for a favorable benefit-risk conclusion.

### 5.2. Depression and Anxiety

Psilocybin has produced rapid reductions in depressive symptoms in treatment-resistant depression and major depressive disorder. A dose-ranging trial found greater improvement after 25 mg than after a very low dose, while placebo-controlled MDD trials reported clinically meaningful short-term differences [43,44,45]. Intravenous DMT also reduced Montgomery-Åsberg Depression Rating Scale scores in a phase IIa randomized trial, supporting proof of concept for a shorter-acting psychedelic intervention [46]. LSD-assisted therapy has shown sustained anxiety reduction in a phase II study, including participants with and without life-threatening illness [47].

The evidence is not uniformly positive. In EPISODE, a three-arm trial comparing psilocybin 25 mg, psilocybin 5 mg, and nicotinamide, the prespecified primary outcome was response on the 17-item Hamilton Rating Scale for Depression at six weeks. The first comparison in the testing hierarchy, 25 mg versus nicotinamide, was not statistically significant, so formal testing of the remaining comparisons did not proceed; several secondary outcomes nevertheless favored the 25-mg dose [9]. A separate 2026 trial reported short-term and late-term symptom changes but also persistent anxiety in two participants [29]. These studies narrow the range of plausible effects and reinforce the need to give prespecified primary outcomes priority over favorable secondary analyses.

Two company-reported phase 3 trials of proprietary psilocybin for treatment-resistant depression met their week-6 primary endpoints, with mean treatment differences of approximately 3.6–3.8 points on the Montgomery-Åsberg Depression Rating Scale [48,49]. Six-month company updates describe maintained benefit and retreatment in subsets. These results are consequential but remain non-peer-reviewed top-line disclosures at the search cut-off. Their magnitude, missing-data handling, multiplicity, safety findings, and blinding assessments cannot be fully evaluated until complete reports and regulatory reviews are available.

### 5.3. Substance Use Disorders and Other Indications

For alcohol use disorder, a randomized trial found fewer heavy-drinking days after psilocybin-assisted psychotherapy than after diphenhydramine-assisted psychotherapy, whereas a small feasibility trial suggested higher abstinence with 25 mg than with 1 mg [50,51]. A living meta-analysis has not established a consistent abstinence benefit across the broader, heterogeneous evidence base [41]. Open-label smoking-cessation studies and early studies in obsessive-compulsive disorder, eating disorders, and demoralization remain hypothesis-generating rather than practice-changing.

### 5.4. Durability and the Transition to Repeat Dosing

Durability is a clinically important but easily overstated claim. Prospective follow-ups after a single or limited course of psilocybin report benefit at 12 months and, in very small self-selected samples, several years [33,34,52]. However, these extensions lack randomized controls, are vulnerable to attrition and subsequent treatment, and cannot separate persistent pharmacological effects from psychotherapy, life changes, or selective follow-up. The strongest conclusion is that some participants maintain benefit for months; the proportion with multi-year drug-attributable remission is uncertain.

Recurrence data point in the same direction. In a long-term follow-up of treatment-resistant depression, many participants experienced a new depressive event within months after single-dose treatment [53]. Nonrandomized repeat dosing was associated with additional symptom reductions, although limitations in sample size and blinding constrain inference [54]. Clinical implementation is therefore likely to create pressure for retreatment or maintenance schedules. Those schedules should not be improvised from single-dose trials because cumulative benefit, optimal interval, and long-term safety are distinct questions. Selected high-impact registered trials, including their principal clinical outcomes, overall clinical impact, and safety signals, are summarized in Table 2.

## 6. Functional Unblinding and the Interpretation of Efficacy

Psychedelic trials face an unusually severe blinding problem because the acute subjective effects are often unmistakable. In the psilocybin-versus-escitalopram trial, the primary six-week difference in self-rated depression was not statistically significant, whereas several secondary outcomes favored psilocybin [55]. The trial neither proved superiority nor equivalence; it demonstrated how conclusions change according to outcome hierarchy and comparator.

A 2026 systematic review of 112 psychedelic randomized trials found that fewer than one-third assessed blinding integrity and that correct treatment guesses were common among both participants and investigators [56]. Functional unblinding can amplify expectancy, alter therapist behavior, affect rater judgment, and produce disappointment in participants assigned to an inactive or minimally active comparator. These mechanisms can bias both subjective symptom outcomes and adverse-event reporting.

An indirect meta-analysis compared psychedelic therapy trials with open-label antidepressant trials in an attempt to equalize the degree of unblinding. It found no statistically significant difference in depression outcomes between the two treatment classes [57]. This result is best understood as a sensitivity analysis, not a head-to-head equivalence trial. Cross-trial comparisons inherit differences in patient populations, treatment intensity, outcome timing, psychotherapy, and publication patterns. The analysis nevertheless shows that claims of categorical superiority over standard antidepressants are not justified by conventional psychedelic-versus-inert-placebo effect sizes alone.

Future trials should measure baseline expectancy; ask participants, therapists, and raters to guess allocation; report blinding indices; and prespecify analyses stratified by treatment guess. Active comparators should reproduce at least some acute sensations without introducing their own therapeutic effect. Where the clinical question is comparative effectiveness rather than regulatory assay sensitivity, adequately powered head-to-head trials against standard treatment are more informative. Independent blinded raters, central adjudication, objective or behavioral outcomes, and measures of functioning and quality of life should complement symptom scales.

## 7. Naturalistic and Population Evidence

Large US population surveys have generally found no association between lifetime classic psychedelic use and greater psychological distress, mental-health treatment, psychotic symptoms, or suicidality; several analyses report inverse associations [58,59,60]. These findings are inconsistent with a large population-wide psychiatric toxicity. They do not establish protection, exclude harm in small vulnerable subgroups, or address dose, recency, frequency, product identity, or context. Healthy-user selection and reverse causation remain plausible.

Experience-focused surveys provide a different view. Among people describing their most difficult psilocybin experience, a substantial minority reported risk of physical harm and a smaller group described enduring adverse effects. Higher estimated dose, longer duration, poor physical comfort, and lack of social support predicted greater difficulty [61]. In a large survey of lifetime users, persistent difficulties were uncommon but included anxiety, negative self-concept, and social disconnection; adverse childhood experiences were strongly associated with risk [62]. A broader cross-sectional comparison of perceived risks and benefits likewise showed substantial heterogeneity rather than uniformly positive or negative outcomes [63].

Prospective observational evidence is beginning to identify effect modification. In a preregistered two-month study, naturalistic psychedelic use was associated with increased psychotic and manic symptom severity only among participants reporting use in an illegal context; psychotic-symptom change varied with use frequency and challenging experiences, while manic change was moderated by histories of schizophrenia or bipolar I disorder [64]. The number of exposed participants with these diagnoses was small, attrition was substantial, and the study cannot establish causality. Nevertheless, it directly contradicts the assumption that context and psychiatric history are peripheral variables.

Real-world clinical cohorts occupy an intermediate position between trials and unsupervised use. A Swiss compassionate-use cohort of 115 patients treated with standardized LSD or psilocybin protocols reported improvements in depression and anxiety over one to three months and few serious complications [65]. The study was retrospective, lacked a control group, and assessed routine clinical data. Such cohorts are valuable for implementation and pharmacovigilance, but effectiveness estimates should not be equated with randomized efficacy.

## 8. Frequency, Repeated Exposure, and Microdosing

### 8.1. What Frequency-Aware Observational Studies Show

The claim that psychedelic-use frequency has never been studied is incorrect. A transcultural survey explicitly classified participants as regular users (more than once every six months), occasional users, or non-users and found lower distress and greater social support among regular users [66]. Another large survey reported that retrospective improvements in depression, anxiety, and wellbeing increased with the number of lifetime exposures before reaching a ceiling, while 13% endorsed at least one harm [67]. In contrast, a survey of recreational users found that high-frequency use and use to cope with negative affect predicted poorer mental health, whereas group use and post-use integration predicted more favorable outcomes [68].

Longitudinal and large cross-sectional datasets remain mixed. A citizen-science cohort observed relative improvement in anxiety and depression among a psychedelic-and-cannabis user cluster across the COVID-19 period, yet increases in drug-use frequency more generally tracked worsening mental health [69]. A global ayahuasca survey found positive associations between number of uses and current mental health, but self-selection, cultural setting, and retrospective reporting limit causal interpretation [70]. Together, these studies show that frequency has been measured, but not in a way that supports a simple dose-frequency response.

The defensible evidentiary gap is narrower and more important: no robust prospective study has assigned or clearly predefined frequency groups, repeatedly measured verified compound and cumulative dose, controlled baseline vulnerability and context, and followed participants long enough to assess psychiatric, cardiovascular, neurocognitive, and functional outcomes. Existing studies use incompatible thresholds, lifetime counts, or broad drug-use clusters; frequent use may be a cause, consequence, or marker of mental-health status. The frequency question is therefore inadequately resolved rather than entirely unasked.

Rapid pharmacodynamic tolerance makes daily full-dose use of classic psychedelics uncommon, but it should not be described as a proven safety mechanism. Users can increase dose, extend dosing intervals, switch compounds, or combine substances. Moreover, tolerance to subjective effects does not necessarily imply tolerance to every receptor-mediated physiological effect. Population rarity also creates a methodological challenge: large cohorts are needed to obtain adequately sized groups with sustained, well-characterized exposure.

### 8.2. Microdosing: Short-Term Evidence and Long-Term Uncertainty

Microdosing usually denotes repeated low or subperceptual doses, often one-tenth or less of a conventional psychedelic dose, taken several times per week. Actual practices vary widely in compound, potency, schedule, and whether subjective effects are truly absent [71]. Unlike occasional macrodosing, this pattern creates repeated exposure over weeks or months and therefore requires its own efficacy and safety evidence.

Placebo-controlled findings have generally been modest. A self-blinding citizen-science study found broad improvement over time in both microdose and placebo groups, with treatment guesses explaining much of the apparent benefit [72]. A preregistered psilocybin study found no reduction in anxiety or depression and no consistent change in emotion processing [73]. Repeated low-dose LSD in healthy adults produced dose-related subjective and physiological effects without clear enhancement of mood or cognition, and a randomized ADHD trial did not establish clinically meaningful efficacy [74,75].

Two 2026 double-blind longitudinal studies of psilocybin microdosing found no reliable improvement in cognitive control, memory, social cognition, wellbeing, or creativity after correction for multiple testing; blinding was comparatively well preserved [76,77]. An eight-week open-label LSD microdosing study in major depression reported marked symptom improvement and described pharmacokinetic exposure, but the absence of placebo control makes expectancy, regression to the mean, and concurrent care inseparable from drug effect [78,79].

Short studies generally report mild, transient adverse effects such as anxiety, headache, increased blood pressure, sleep disturbance, or cognitive interference. A 2025 systematic review identified heterogeneous and often incomplete safety reporting and little evidence beyond short durations [80]. Thus, microdosing is no longer unstudied, but evidence for benefit is weak and evidence for sustained safety remains inadequate.

### 8.3. The 5-HT2B Receptor and Valvular-Heart-Disease Hypothesis

The principal mechanistic concern for long-term microdosing is chronic agonist activity at the serotonin 2B (5-HT2B) receptor. Persistent 5-HT2B stimulation is an established pathway for drug-induced valvular fibroplasia with agents such as fenfluramine and pergolide. Several psychedelics and MDMA have measurable 5-HT2B activity, but receptor potency, functional efficacy, active-metabolite exposure, dose, and duration vary. Extrapolation from known valvulopathogens therefore supports a plausible hazard hypothesis, not a demonstrated clinical syndrome [81,82,83].

The first large epidemiological analysis used All of Us Research Program data and found that lifetime hallucinogen use was associated with slightly higher adjusted odds of a valvular-heart-disease diagnosis [84]. The exposure variable combined heterogeneous substances and contained no frequency, dose, duration, or microdosing information; outcomes were diagnostic codes rather than standardized echocardiography. Residual confounding is likely. The study justifies further investigation but cannot determine whether sustained microdosing damages heart valves.

A proportionate response is targeted surveillance rather than alarm. Prospective microdosing cohorts should record verified dose and frequency, cumulative exposure, concomitant serotonergic agents, blood pressure, cardiac symptoms, and baseline and follow-up echocardiography. Nested case-control analyses within registries could then determine whether any valvular signal is concentrated by compound, duration, or susceptibility.

### 8.4. Repeated Recreational MDMA Exposure Is a Distinct Problem

Repeated recreational MDMA use should not be pooled mechanistically with occasional supervised classic-psychedelic dosing. High or repeated MDMA doses can produce serotonergic neurotoxicity in animal models, with strong dependence on dose, redosing, hyperthermia, and species [85]. Human studies of heavy users report learning and memory differences and altered serotonergic markers, but the literature is confounded by polydrug use, sleep deprivation, product adulteration, premorbid traits, and uncertain lifetime dose. A recent systematic review continues to rate the long-term neurocognitive evidence as low quality [86,87].

The clinical MDMA regimen—known product, limited sessions, controlled temperature, medical monitoring, and no concurrent stimulants—differs sharply from repeated weekend use with redosing, heat, exertion, and uncertain tablet content [88]. Evidence from one exposure pattern should not be used to reassure or alarm about the other. Long-term registries of therapeutic MDMA should nevertheless include cognition, sleep, mood instability, and subsequent nonmedical use because current trials are not large enough to exclude uncommon cumulative effects.

## 9. Risk Modifiers and the Therapeutic Frame

The most defensible risk modifiers are those supported by converging clinical practice, pharmacology, and observational evidence. Personal or family history of psychotic or bipolar disorder remains a central exclusion criterion; cardiovascular disease and older age increase concern about autonomic activation; and medication interactions may alter exposure or physiological response [3,4]. Dose and prolonged acute distress predict difficult experiences. Psychiatric history, adverse childhood experiences, younger age, and trait absorption have been associated with persistent psychological or perceptual symptoms [36,62].

Context is not merely background. Preparation, trust, monitor competence, social support, and post-session integration may influence both acute safety and reported benefit [14,15]. However, observational correlations between mystical-type experience, emotional breakthrough, alliance, and outcome are vulnerable to common-method bias and post-randomization confounding. An intense positive experience may be a mediator, a marker of effective drug exposure, or a consequence of early improvement. Trials should avoid turning these correlates into mandatory therapeutic narratives without experimental testing.

The psychotherapeutic component is also heterogeneous. Manuals, therapist training, session number, allegiance, and integration practices differ across programs, while many trials are not designed to isolate psychotherapy from drug effects. Standardized reporting of therapist qualifications, protocol adherence, boundary violations, and adverse psychological processes is essential. The safety profile of psychedelic-assisted therapy belongs to the entire intervention, but that proposition should not obscure compound-specific pharmacological risks.

## 10. Regulatory and Implementation Context

Australia’s authorized-prescriber pathway provides controlled clinical access to unapproved MDMA and psilocybin products for narrowly specified indications. The TGA requires specialist psychiatric oversight, an approved clinical protocol, ethics review, supervised administration, and compliance with product-quality and reporting obligations [5,6]. Because the products are unapproved, access should be understood as a monitored exception rather than evidence that efficacy and safety are settled.

The FDA’s 2024 complete response letter for midomafetamine capsules highlights the regulatory importance of functional unblinding, trial conduct, adverse-event ascertainment, and durability [42]. For psilocybin, two positive phase 3 company announcements are now available, and the sponsor has reported a rolling regulatory submission; full peer-reviewed reports and regulatory analyses remain necessary before benefit-risk conclusions can be independently evaluated [48,49].

Implementation schemes create a scientific opportunity. Mandatory registries can capture less highly selected patients, retreatment, protocol variation, and events that occur after trial-like follow-up ends. Minimum datasets should include indication, compound verification, dose, session number, cumulative exposure, co-medication, cardiovascular measures, acute psychological events, suicidality, mania, psychosis, perceptual symptoms, functioning, and all subsequent mental-health treatment. Without standardized longitudinal capture, real-world access will generate volume without reliable evidence.

## 11. Research Priorities

The priority questions and corresponding minimally informative designs are summarized in Table 3.

According to these priorities, adverse-event measurement should be harmonized. Investigators should distinguish adverse events from adverse drug reactions; define onset and follow-up windows; report severity, expectedness, treatment, resolution, and functional consequences; and adjudicate causality independently when feasible. HPPD-type symptoms should be measured with instruments that separate transient visual phenomena from distress and impairment. Suicidality should be reported by baseline risk, time relative to dosing, and treatment allocation rather than collapsed into an undifferentiated event count.

Statistical plans should treat exposure as time-varying where appropriate and model compound, dose, frequency, and cumulative exposure separately. Propensity scores cannot remove unmeasured self-selection, but target-trial emulation, negative-control outcomes, within-person analyses, and sensitivity analyses can improve observational inference. Data linkage to health records and echocardiography can reduce sole reliance on retrospective self-report. Registries and trials should make de-identified protocols and analytic code available to permit independent reanalysis.

## 12. Conclusions

For screened adults receiving one to three supervised doses, classic psychedelics and MDMA-assisted therapy have a generally manageable acute safety profile characterized by transient psychological and autonomic effects. Serious drug-related events are uncommon but real, and recent reports of persistent anxiety, suicidality signals, and HPPD preclude absolute reassurance. The absence of persistent psychosis in most modern trials is partly a consequence of careful exclusion and monitoring.

Clinical outcome evidence is strongest for MDMA-assisted therapy in PTSD and for short-term psilocybin-based treatment in depression, but certainty is reduced by functional unblinding, small or selected samples, heterogeneous psychosocial interventions, and inconsistent comparators. Recent negative and equivocal primary outcomes, together with non-peer-reviewed phase 3 announcements, argue for calibration rather than either dismissal or therapeutic exceptionalism.

Frequency has been studied, but mostly with retrospective, cross-sectional, or broadly clustered measures. The unanswered question is whether well-characterized repeated exposure changes long-term psychiatric, cardiovascular, neurocognitive, or functional outcomes. Microdosing trials now provide useful short-term evidence but little consistent support for broad cognitive or emotional benefits. They do not resolve sustained safety concerns. A plausible 5-HT2B-mediated valvular hazard remains unconfirmed in humans.

Future efficacy research should distinguish short-term benefit under intensive trial support from comparative effectiveness and durability in routine care. Confirmatory trials should use psychoactive or dose-ranging comparators, centrally blinded raters, repeated assessments of expectancy, and outcomes that extend beyond symptom scores to include remission, functioning, and quality of life. Relapse, rescue treatment, and retreatment should be prespecified, with randomized maintenance strategies and blinded follow-up long enough to define optimal interdose intervals and cumulative treatment burden. These priorities are consistent with the FDA’s final 2026 guidance, which recommends assessment at 12 weeks, longer-term blinded follow-up (typically 12 months), and prespecified retreatment criteria [89].

Future trials should also separate the intervention into its active components. Factorial designs can estimate the contributions of drug dose, preparation, in-session monitoring, post-session psychotherapy, expectancy, therapist training, and protocol fidelity. Safety surveillance must extend beyond the dosing day to delayed suicidality, mania or psychosis, persistent anxiety and perceptual symptoms, cardiovascular outcomes, cognitive and functional change, medication interactions, and later nonmedical use. Serial echocardiography is particularly relevant when sustained exposure involves compounds with 5-HT2B agonism [89].

Taken together, this agenda would move the field from molecule-level generalizations to compound-, regimen-, person-, and context-specific claims. Progress should be judged by whether independent evidence can identify who benefits, who is harmed, how long benefit persists, what follow-up or retreatment is required, and whether gains important to patients justify the clinical and societal costs.

## Figures and Tables

**Table 1:** Exposure patterns, evidence maturity, and principal uncertainties.

Principal Uncertainty	Evidence Base	Person and Context	Typical Dose and Frequency	Exposure Pattern
Rare events, generalizability, expectancy and psychotherapy	Multiple RCTs, meta-analyses, and follow-ups	Screened patients, preparation, monitoring, follow-up	Known moderate-to-high dose; usually 1–3 sessions separated by weeks	**Supervised therapeutic macrodosing**
Optimal interval, cumulative benefit and risk, dependency on support model	Small trials, open-label, company phase 3 updates	Clinical care or extension studies, variable retreatment rules	Additional full-dose sessions over months or years	**Repeated or maintenance therapeutic dosing**
Selection, recall, confounding, uncertain dose and compound	Cross-sectional surveys and prospective cohorts	Unscreened, variable setting, substance verification, and co-use	Self-selected dose; typically infrequent	**Occasional naturalistic use**
Direction of causality, psychiatric vulnerability, functional outcomes	A few frequency-aware observational studies	Unscreened, heterogeneous motives and environments	Monthly or more often, cumulative dose rarely quantified	**Frequent full-dose naturalistic use**
Long-term cardiovascular, psychiatric, and neurocognitive safety, dose verification	Short randomized studies and observational cohorts	Usually self-directed, everyday activities continue	Subperceptual or low dose, commonly 2–4 times weekly for weeks or months	**Sustained microdosing**
Separating MDMA effects from dose, hyperthermia, polydrug use, and premorbid differences	Observational neurocognitive studies and animal models	Heat, exertion, sleep loss, variable product purity	Repeated high or uncertain doses, often with redosing and polydrug use	**Heavy recreational MDMA exposure**

**Table 2:** Selected high-impact registered clinical trials of psychedelic and MDMA-assisted interventions: outcomes, clinical impact, and safety signals.

General Impact and Interpretation	Observed Safety Issues	Principal Clinical Outcome	Design, Sample, Comparator, and Trial ID	Intervention and Indication
First pivotal phase 3 efficacy signal, supported the confirmatory and regulatory program.	Transient autonomic and expected MDMA effects. All three serious suicidality events occurred in the placebo group.	CAPS-5 change: −24.4 vs. −13.9 (d = 0.91); 67% vs. 32% of completers no longer met PTSD criteria.	MAPP1, phase 3 RCT, *n* = 90, placebo plus identical therapy; NCT03537014 [7]	**MDMA-assisted therapy/severe PTSD**
Replicated the efficacy in a more diverse sample.	Severe AEs occurred in 9.4% vs. 3.9%. Muscle tightness, nausea, appetite loss, sweating, mild cardiac symptoms, and transient blood-pressure and pulse increases were observed.	CAPS-5 least-squares mean difference: −8.9 (95% CI, −13.70 to −4.12; *p* < 0.001; d = 0.70); 71.2% vs. 47.6% no longer met PTSD criteria.	MAPP2, confirmatory phase 3 RCT, *n* = 104, placebo plus identical therapy; NCT04077437 [8]	**MDMA-assisted therapy/moderate-to-severe PTSD**
Multicenter dose-ranging trial, identified a 25-mg short-term signal.	AEs occurred in 77%, chiefly headache, nausea, and dizziness. Suicidal ideation, behavior, or self-injury occurred in all dose groups, with serious events in the higher-dose arms.	MADRS difference for 25 mg vs. 1 mg was −6.6 (95% CI, −10.2 to −2.9; *p* < 0.001); 10 mg was nonsignificant, and sustained response at week 12 was not clearly superior.	COMP360 phase 2b dose-ranging RCT, *n* = 233, 25 mg or 10 mg vs. 1 mg; NCT03775200 [43]	**Psilocybin/treatment-resistant depression**
First direct SSRI comparison showed why primary-outcome hierarchy and functional unblinding affect interpretation.	AE incidence was 87% vs. 83%. Transient headache was most common with psilocybin.	The prespecified QIDS-SR-16 difference: −2.0 (95% CI, −5.0 to 0.9; *p* = 0.17), superiority was not demonstrated, several unadjusted secondary outcomes favored psilocybin.	Phase 2 RCT, *n* = 59, two 25-mg sessions vs. two 1-mg sessions plus 6 weeks of escitalopram; NCT03429075 [55]	**Psilocybin/major depressive disorder**
Placebo-controlled efficacy, effect magnitude remains vulnerable to unblinding.	AEs were more frequent with psilocybin: headache (66%), nausea (48%), and visual effects (44%).	At day 43, the MADRS difference was −12.3 (95% CI, −17.5 to −7.2; *p* < 0.001), with improved disability and sustained response in 42% vs. 11%.	Multisite phase 2 RCT, *n* = 104, 25 mg vs. niacin with identical support; NCT03866174 [45]	**Psilocybin/major depressive disorder**
Negative primary trial, favorable symptom-change analyses remain exploratory.	Dosing-day suicidal ideation was 4% vs. 1–2%. Two serious adverse reactions followed 25 mg, including one case of HPPD.	Week-6 HAMD-17 response was 17.0%, 12.5%, and 10.6%, the 25-mg vs. nicotinamide primary comparison was nonsignificant (OR, 1.73; 95% CI, 0.53–6.23; *p* = 0.19).	EPISODE triple-blind phase 2b RCT, *n* = 144, 25 mg vs. 5 mg vs. nicotinamide; NCT04670081 [9]	**Psilocybin/treatment-resistant depression**
Small efficacy signal with longer follow-up, post-session anxiety can require sustained medical support.	Most AEs were transient and mild to moderate, two psilocybin recipients developed persistent severe anxiety requiring medical attention.	The day-8 MADRS difference was −7.27 (95% CI, −12.89 to −1.65; *p* = 0.01); differences persisted through day 42 but not day 365.	PSIPET RCT, *n* = 35, 25 mg vs. niacin plus five support sessions; NCT04630964 [29]	**Psilocybin/recurrent major depressive disorder**
Controlled addiction trial, cannot isolate the drug from intensive psychotherapy.	Headache, anxiety, and nausea were common, two participants received diazepam, one suicidal ideation.	Heavy-drinking days were 9.7% vs. 23.6% (95% CI, 3.0–24.7; *p* = 0.01).	RCT, *n* = 95, two psilocybin vs. diphenhydramine sessions plus manualized psychotherapy; NCT02061293 [50]	**Psilocybin-assisted psychotherapy/alcohol use disorder**
Sponsor-reported positive phase 3 primary endpoint, pending peer review and regulatory analysis.	Headache, nausea, and visual hallucination, 11 treatment-emergent severe AEs occurred in 8 participants receiving 25 mg.	Week-6 MADRS difference: −3.6 (95% CI, −5.7 to −1.5; *p* < 0.001).	COMP005 phase 3 RCT, *n* = 258, single 25-mg dose vs. placebo; NCT05624268 [48]	**COMP360 psilocybin/treatment-resistant depression**
Sponsor-reported positive phase 3 result, durability effects.	Nausea, headache, anxiety, visual hallucination, six severe AEs occurred in six 25-mg recipients.	Week-6 MADRS difference for 25 mg vs. 1 mg: −3.8 (95% CI, −5.8 to −1.8; *p* < 0.001), reported durability through week 26.	COMP006 phase 3 RCT, *n* = 581, two 25-mg, 10-mg, or 1-mg doses; NCT05711940 [49]	**COMP360 psilocybin/treatment-resistant depression**
Controlled LSD-anxiety signal, crossover limits the primary analysis.	AEs occurred in 19%, one severe acute anxiety and a delusional reaction. Cardiovascular activation was transient, and no HPPD occurred.	Week-16 STAI-G difference: −16.2 (95% CI, −27.8 to −4.5; *p* = 0.007).	Randomized phase 2 crossover trial, *n* = 42, two 200-microgram sessions vs. placebo; NCT03153579 [47]	**LSD-assisted therapy/anxiety with or without life-threatening illness**
Endpoint signal does not establish robust efficacy and underscores the difficulty of delayed-event attribution.	Three delayed hospitalizations (two low-dose, one high-dose), a later suicide after (low-dose-arm).	IDS-SR difference: −7.9 (95% CI, −16.0 to 0.3; *p* = 0.059), IDS-C difference: was −9.2 (*p* = 0.023).	Active low-dose-controlled RCT, *n* = 61, 100 plus 200 micrograms vs. 25 plus 25 micrograms; NCT03866252 [28]	**LSD-assisted therapy/major depressive disorder**
Antidepressant proof of concept for a short-duration psychedelic intervention.	Infusion-site pain, nausea, and transient anxiety.	Week-2 MADRS difference: −7.35 (95% CI, −13.62 to −1.08; *p* = 0.023), improvement persisted through 3 months.	SPL026 phase IIa RCT, *n* = 34, 21.5 mg IV DMT vs. placebo, followed by open-label DMT; NCT04673383 [46]	**Intravenous DMT/major depressive disorder**

Notes: Trials were selected for phase, sample size, clinical influence, informative comparators, or important safety signals; the list is not exhaustive. Negative differences favor the active intervention on symptom-severity scales. Abbreviations: AE, adverse event; CAPS-5, Clinician-Administered PTSD Scale for DSM-5; DMT, N,N-dimethyltryptamine; HAMD-17, 17-item Hamilton Depression Rating Scale; HPPD, hallucinogen persisting perception disorder; IDS-C/SR, clinician-/self-rated Inventory of Depressive Symptomatology; LSD, lysergic acid diethylamide; MADRS, Montgomery-Åsberg Depression Rating Scale; PTSD, post-traumatic stress disorder; QIDS-SR-16, 16-item Quick Inventory of Depressive Symptomatology-Self-Report; STAI-G, State-Trait Anxiety Inventory-Global.

**Table 3:** Priority questions and minimally informative designs.

Why Current Evidence is Insufficient	Essential Measurements	Minimum Informative Design	Priority Question
Cross-sectional and retrospective studies use incompatible thresholds and cannot resolve direction of causality	Verified compound when possible, dose, cumulative exposure, context, baseline vulnerability, psychiatric and functional outcomes	Prospective cohort with predefined frequency strata and repeated exposure assessment for at least 12–24 months	**Does frequent naturalistic use alter long-term health?**
No large-scale studies link microdosing to standardized valve imaging	Echocardiography, ambulatory blood pressure, ECG, symptoms, 5-HT2B-relevant co-medication, cumulative dose	Prospective exposure-defined cohort or pragmatic trial with baseline and serial cardiac assessment	**Is sustained microdosing cardiotoxic?**
Single-dose trials and open-label retreatment cannot define optimal interval and long-term benefit	Time to relapse, functioning, quality of life, adverse events, cumulative dose, treatment burden	Randomized retreatment/maintenance strategy trial	**What is the value of maintenance macrodosing?**
Inert comparators create asymmetric unblinding, meta-analyses cannot establish equivalence	Expectancy, allocation guess, blinded symptom ratings, function, health-care use, adverse events	Head-to-head comparative-effectiveness trial with measured blinding and independent raters	**How much efficacy remains under matched expectancy?**
Trials exclude higher-risk groups and naturalistic studies contain few well-characterized cases	Family history, trauma, age, medication, mania, psychosis, suicidality, perceptual disorder	Large registry-linked cohort, monitoring vulnerable groups	**Which patients are at elevated psychiatric risk?**
Routine-care cohorts are small, retrospective, and heterogeneous	Protocol fidelity, therapist variables, repeat dosing, serious events, boundary violations, patient-reported harms	Mandatory jurisdictional registry with active pharmacovigilance and long follow-up	**How safe is routine clinical implementation?**

## Data Availability

No new data were created or analyzed in this study. Data sharing is not applicable to this article.
